# Sound generator associated with the counseling in the treatment of tinnitus: evaluation of the effectiveness^☆^

**DOI:** 10.1016/j.bjorl.2016.03.021

**Published:** 2016-11-11

**Authors:** Andressa Vital Rocha, Maria Fernanda Capoani Garcia Mondelli

**Affiliations:** a Faculdade de Odontologia de Bauru, Universidade de São Paulo (USP), Bauru, SP, Brazil; b Department of Audiology and Speech Pathology, Faculdade de Odontologia de Bauru, Universidade de São Paulo (USP), Bauru, SP, Brazil

**Keywords:** Tinnitus, Hearing loss, Hearing aid, Zumbido, Perda auditiva, Aparelho auditivo

## Abstract

**Introduction:**

The relations between the tinnitus and the hearing loss are due to the sensory deprivation caused by hearing loss, since this is followed by the functional and structural alteration of the auditory system as a whole. The cochlear lesions are accompanied by a reduction in the activity of the cochlear nerve, and the neural activity keeps increased in mainly all the central auditory nervous system to compensate this deficit.

**Objective:**

This study aimed to verify the effectiveness of the sound generator (SG) associated with the counseling in the treatment of the tinnitus in individuals with and without hearing loss regarding the improvement of the nuisance through Tinnitus Handicap Inventory (THI) and Visual Analogue Scale (VAS).

**Methods:**

The sample consisted of 30 individuals of both genders divided into two groups: Group 1 (G1) was comprised of 15 individuals with tinnitus and normal hearing, adapted to SG; Group 2 (G2) was comprised of 15 individuals with complaints of hearing acuity and tinnitus, adapted with SG and an individual hearing aid device (HA). Both groups underwent the following procedures: anamnesis and history of complaint, high frequency audiometry (HFA), imitanciometry, acuphenometry with the survey of psychoacoustic pitch and loudness thresholds and application of the tools THI and VAS. All of them were adapted with HA and Siemens SG and participated in a session of counseling. The individuals were assessed in three situations: initial assessment (before the adaptation of the HA and SG), monitoring and final assessment (6 months after adaptation).

**Results:**

The comparison of the tinnitus nuisance and handicap in the three stages of assessment showed a significant improvement for both groups.

**Conclusion:**

The use of the SG was similarly effective in the treatment of the tinnitus in individuals with and without hearing loss, causing an improvement of the nuisance and handicap.

## Resumo

**Introdução:**

As relações entre o zumbido e a perda de audição advêm da privação sensorial causada pela perda auditiva, uma vez que esta é seguida pela alteração estrutural e funcional do sistema auditivo no seu conjunto. As lesões cocleares são acompanhadas por uma redução da atividade do nervo coclear e a atividade neuronal mantém-se aumentada em principalmente todo o Sistema Nervoso Auditivo Central para compensar este déficit.

**Objetivo:**

Este estudo teve como objetivo verificar a eficácia do Gerador de Som (GS) associado ao aconselhamento no tratamento do zumbido em indivíduos com e sem perda auditiva em relação à melhora do incômodo por meio do *Tinnitus Handicap Inventory* (THI) e Escala Analógica Visual (VAS).

**Método:**

A amostra consistiu em 30 indivíduos de ambos os sexos, divididos em dois grupos: Grupo 1 (G1) foi composto por 15 indivíduos com zumbido e audição normal, adaptados ao GS; o Grupo 2 (G2) consistiu em 15 indivíduos com queixas de acuidade auditiva e zumbido, adaptado com GS e um aparelho auditivo individual (AA). Ambos os grupos foram submetidos aos seguintes procedimentos: anamnese e história da queixa, Audiometria de Alta Frequência (AAF), imitanciometria, acufenometria com o levantamento de limiares psicoacústicos de Frequência e Altura e aplicação das ferramentas THI e VAS. Todos eles receberam adaptação de AA e GS Siemens e participaram de uma sessão de aconselhamento. Os indivíduos foram avaliados em três situações: Avaliação Inicial (antes da adaptação do AA e GS), Monitoramento e Avaliação Final (6 meses após a adaptação).

**Resultados:**

A comparação do incômodo do zumbido e deficiência nas três fases de avaliação mostrou uma melhora significativa nos dois grupos.

**Conclusão:**

O uso de GS foi igualmente eficaz no tratamento do zumbido nos indivíduos com e sem a perda de audição, causando uma melhora da perturbação e da deficiência.

## Introduction

The relations between the tinnitus and the hearing loss are due to the sensory deprivation caused by hearing loss, since this is followed by the functional and structural alteration of the auditory system as a whole. The cochlear lesions are accompanied by a reduction in the activity of the cochlear nerve, and the neural activity keeps increased in mainly all the Central Auditory Nervous System (CANS) to compensate this deficit. The increase in the activity of the CANS characterizes a hyperactivity of the nervous structures that result in a “neural noise”. This noise can be codified by the own nervous system generating the perception of the tinnitus.^1^

The theories suggest the tinnitus is caused by a sequence of central changes that are triggered by the decrease in the afferent of the sound stimulus. A prediction resulting from this theory is that the compensation of such afferent may be a way of preventing or reversing the changes in the CANS badly adapted, which underlies the tinnitus. The acoustic stimulation, for example, could compensate this decreased afference.^2^

The acoustic therapy is performed with the insertion of sound enrichment in the individual's daily life, and it aims to provide relief from tinnitus. In less specific interventions, the individual may be advised to use strategies, such as: insert background music during daily activities, use sounds of relaxation, listen to music with earphones, use pillows with speakers, use water cascades, use sound generators in the tinnitus level and conventional Hearing Aids (HA).^3^ The HA consists of the amplification of environmental sounds – both mask the tinnitus and help in habituation by sound enrichment, the SG produces the wideband noise, songs or any other spectrally modified type of sound.^4^

There is a need of presenting scientific evidence in the tinnitus treatments to help the professionals in decision-making, therapeutic handling of patients and development of clinical guidelines that would direct the assessments and interventional approaches in individuals with and without hearing loss.

This study aimed to verify the effectiveness of the sound generator (SG) associated with the counseling in the treatment of the tinnitus in individuals with and without hearing loss regarding the improvement of the nuisance and handicap.

## Methods

The study was designed as a prospective cohort nonrandomized clinical trial, and it was carried out at the Speech-Language Pathology and Audiology Clinic, with the approval of the Institution Research Ethics Committee, under the n° 18001213.4.0000.5417.

A number of 30 subjects of both genders were eligible for the inclusion in the sample of the study. They reported a complaint of bilateral chronic tinnitus and were divided into two groups. Group 1 (G1) was comprised of 15 individuals with normal hearing and Group 2 (G2) was comprised of 15 individuals with a diagnosis of symmetrical bilateral sensorineural hearing loss from a mild to moderate degree.

### Group 1

G1 was comprised of 15 individuals of both genders (80% female and 20% male), without a complaint of auditory acuity. The average age was of 55.87 years, with a standard deviation of 10.27. The most frequent tinnitus were whistle (*n* = 3), pressure cooker (*n* = 1), mosquito (*n* = 1), bell (*n* = 1), cricket (*n* = 2), grasshopper (*n* = 1), waterfall (*n* = 1), wheezing (*n* = 2), feedback (*n* = 1), bee (*n* = 1) and butterfly (*n* = 1). All symptoms were referred to bilaterally (*n* = 15).

Regarding the hearing, the mean of the hearing thresholds obtained for this group was of 15.33 dB – with a standard deviation of 5.33 for the right ear, and mean of 15.50 dB – with a standard deviation of 5.08 for the left ear.

### Group 2

G2 was comprised of 15 individuals of both genders (53.33% female and 46.66% male), with a complaint of auditory acuity. The average age was 63.6 years, with a standard deviation of 7.61.

**Figure 1:**
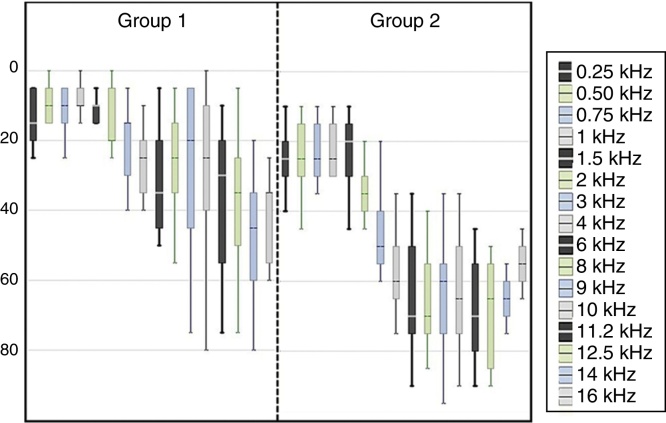
Values of mean, maximum and minimum audiometric thresholds of the right ear (RE) in the stages initial assessment, monitoring and final assessment of the groups G1 and G2.

**Figure 2:**
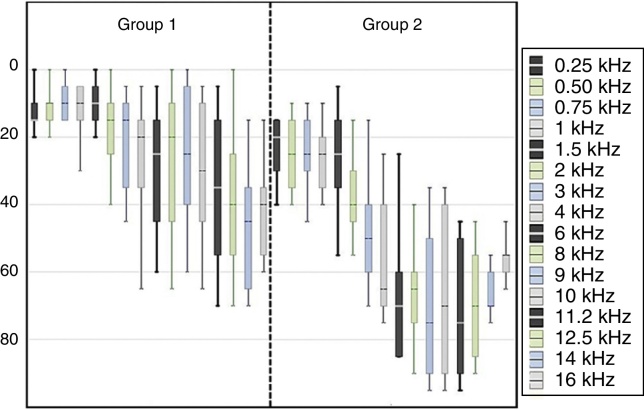
Values of mean, maximum and minimum audiometric thresholds of the left ear (LE) in the stages initial assessment, monitoring and final assessment of the groups G1 and G2.

The mean of the hearing thresholds obtained was of 37.77 dB – with a standard deviation of 7.52 for the right ear, and mean of 35.83 dB – with a standard deviation of 11.21 for the left ear. Figure 1, Figure 2 bring the thresholds obtained for the conventional audiometry and high frequency audiometry for the right and left ears.

Regarding the degree of hearing loss, 9 individuals presented mild degree and 6 moderate degree, equivalent to 60% and 40%, respectively.

The study was comprised of three stages: initial assessment – stage in which the patient had a complaint of tinnitus without intervention. Monitoring (3 months) and final assessment (6 months).

After the otorhinolaryngological evaluation, the individuals underwent anamnesis and Visual Inspection of the External Acoustic Meatus (EAM) to verify the occurrence of some impediment of the middle and/or outer ear. Afterwards, they underwent the hearing assessment including conventional pure tone audiometry and HFA. The extended frequencies evaluated from 8 kHz were 9, 10, 12.2, 12.5, 14 and 16 kHz.

The questionnaire Tinnitus Handicap Inventory (THI) was applied in all stages of the study; it was translated and validated for the Brazilian Portuguese^5^ and assesses the tinnitus handicap by measuring its general nuisance and domains: functional, emotional and catastrophic. The highest total score possible corresponds to 100 points, and it is believed to show a maximum impairment of the patient's daily life due to the tinnitus.

The VAS can also measure the nuisance of the tinnitus for the individual. The scale consists of a “ruler” with graphic representations equivalent to the grades 0–10. The use of the image produces a visual reinforcement that can be remembered by the patient.

The measuring of the tinnitus was performed through the acuphenometry, with the survey of Pitch and Loudness similar to the individual's sensation. This method is subjective and comprises a set of audiological techniques to try to find a psychoacoustic threshold the closest possible to the patient's tinnitus at the moment the symptom occurrence is. The patients were told to signal raising one of the hands whenever the sign presented was the closest to their tinnitus.

Initially, the Pitch (sensation of frequency) of the tinnitus was investigated through a pure tone or narrowband noise, depending on the characterization of the symptom presented by the individual. The stimuli were presented 5 dB higher than the audiometric thresholds. Afterwards, the Loudness (sensation of intensity) was investigated in the previously estimated frequency of the tinnitus and in its audibility threshold. The signal was increased in steps of 5 dB until the patient reported to be equivalent to the system. The thresholds were retested, and we found the mean of the values obtained in the two times in which the “selected” stimuli intensities were presented. In the occurrence of the tinnitus suppression (partial or total), the repetitions were not measured, through the absence of the symptom.

The individuals were adapted to the HA mini retroauricular open fitting, Life, Siemens, with silicone eartips and a thin tube, according to each participant's audiological characteristics, physical and acoustic needs.

The HA presented the conventional resources of amplification associated with the SG (Programmable Tinnitus Control algorithm) with wideband noise (white noise), from 250 Hz to 8 kHz, which can be used as an amplifier, sound generator or both.

Thus, the groups were adapted as follows:

Group 1 (normal hearing and tinnitus): bilateral adaptation with “sound generator” activated, considered as “only” adaptation.
Group 2 (hearing loss and tinnitus): bilateral adaptation with the “sound generator” and “amplification” activated to overcome hearing loss, and considered as a “combined” adaptation.

The SG adjustments for the groups were performed in an ascendant way. Thus, the intensity of the noise was gradually increased until the patient signaled the comfort and audibility point was met. The “mixing point” was verified in several situations as some patients reported the mixture of both sounds.

To ensure the effective use of the HA and verify the effectiveness of the sound therapy, only software was activated: “universal”. G1 with only the SG and G2 with the HA and the SG associated providing stimuli concomitantly. The individual were told to use the HA for a minimum period of 8 h a day, taking it out to shower and sleep. The effective use was verified through the activation of the data logging algorithm.

To verify the amplifications, patients of the G2 had the measures taken with probe microphone, an objective method that expresses the real quantity of amplification provided by the HA in the patient's conduit. We selected the prescriptive method developed by the National Acoustic Laboratories – NAL NL1, for non-linear HA, compatible with the equipment rule, in which the measurements were performed to verify the effectiveness of the amplification.

At the time of adapting the HA, a counseling session was carried out. This session addressed issues regarding hearing physiology, pathophysiology of tinnitus and hearing, using simple language and digital material with some illustrative images proposed^6^ – authors responsible for the scientific basis of the counseling associated with the fitting of the Siemens HA used in this study.

From these guidelines and awareness, the research participants obtained the audibility and comfort point of the SG, based on the auditory perception of the patient, finishing the programming of the hearing aids.

## Methods and statistical analysis

The analysis of the results was carried out based on the inductive or inferential statistics. All statistical procedures were performed in the software 5.1 Statistica (Stat Soft Inc., Tulsa, USA), with a significance level equal to 5%. For the descriptive analysis, we used the mean and the standard deviation of all numerical variables. For the inferential analysis, we performed the analysis of variance of the measures repeated in both criteria (ANOVA) to verify the possible significance in the treatment of the groups isolated. After the ANOVA and the finding of a significant difference between treatments, we used the test of multiple comparisons to verify the magnitude of such differences: Tukey's test.

## Results

Results of the THI in the stages initial assessment, monitoring and final assessment for both groups are described in Table 1.

**Table 1:** Values of mean and SD of the answers obtained for the THI – total score, functional, emotional and catastrophic categories in the three stages of assessment.

THI	Mean ± SD	
	G1	G2
*Total score*		
IA	66.66 ± 12.27^a^	66.4 ± 13.79^a^
MO	22.13 ± 16.96^b^	18.66 ± 12.45^b^
FA	11.6 ± 10.03^c^	10.6 ± 12.88^b^

*Functional*		
IA	36.66 ± 10.32^a^	34.2 ± 7.38^a^
MO	9.86 ± 8.33^b^	10.8 ± 6.53^b^
FA	5.46 ± 5.26^b^	6.4 ± 6.97^b^

*Emotional*		
IA	16.66 ± 6.17^a^	18.8 ± 6.36^a^
MO	5.6 ± 5.19^b^	3.6 ± 4.61^b^
FA	2.53 ± 3.81^b^	2.93 ± 4.58^b^

*Catastrophic*		
IA	12 ± 3.46^a^	14.13 ± 5.47^a^
MO	4.8 ± 3.76^b^	4.26 ± 2.49^b^
FA	2.53 ± 2.35^b^	2.93 ± 3.01^b^

Different superscript letters in the same category indicate statistically significant difference in the comparison between groups (repeated measures ANOVA and Tukey, *p* < 0.05 statistically significant).

SD, standard deviation; IA, initial assessment; MO, monitoring; FA, final assessment.

Results of the VAS in the stages: initial assessment, monitoring and final assessment for both groups are described in Table 2.

**Table 2:** Values of mean and SD of the answers obtained for the VAS in the three stages of assessment.

VAS	Mean ± SD	
	G1	G2
IA	8.66 ± 1.34^a^	8.0 ± 1.19^a^
MO	2.93 ± 1.79^b^	3.4 ± 2.02^b^
FA	1.8 ± 1.78^b^	1.66 ± 1.95^c^

Different superscript letters in the same category indicate statistically significant difference in the comparison between groups (repeated measures ANOVA and Tukey, *p* < 0.005 statistically significant.

SD, standard deviation; IA, initial assessment; MO, monitoring; FA, final.

The psychoacoustic thresholds of the acuphenometry results in the first stage of the study for both groups are described in Table 3. The psychoacoustic thresholds were not assessed in the stages of monitoring and final assessment of the patients without tinnitus at the moment of the care, from a partial or total suppression of the symptom.

**Table 3:** Values of mean and SD of the answers obtained in the acuphenometry in the initial assessment.

Acuphenometry	Mean ± SD	
	G1	G2
*Pitch of the tinnitus*		
RE	8.16 ± 2.92	6.73 ± 1.94
LE	8.44 ± 2.71	6.86 ± 1.95

*Loudness of the tinnitus (dBSL)*		
RE	−1.33 ± 14.69	1.33 ± 13.07
LE	−2.66 ± 14.86	7.66 ± 10.66

SD, standard deviation; RE, right ear; LE, left ear; Hz, hertz; dBSL, decibel sensation level.

## Discussion

Professionals who work in the hearing area have experienced an increase in the number of individuals with tinnitus – with or without hearing loss – that look for an effective intervention.

The configuration of the audiometric curves varied (Figure 1, Figure 2) due to the proposal of comprising a group with hearing thresholds within the normal range (G1), based on the conventional audiological diagnosis, and a group with lowered hearing thresholds from 26 dB HL to approximately 60 dB HL (G2). The results of the HFA showed the occurrence of audiometric curves with lowered thresholds from 8 kHz for both groups, suggesting the beginning of a cochlear lesion even in the individuals diagnosed with a normal hearing. Such data corroborates the studies that indicate the origin of the tinnitus from the reduction or absence of afferent in the CANS.^1^, ^2^, ^7^

The performance of the HFA occurs in the hearing monitoring in individuals at risk of developing hearing alterations caused by endogenous or exogenous factors. Study^8^ observed the frequencies from 4 kHz to 6 kHz in the conventional audiometry, and 14 kHz and 16 kHz in the HFA are more affected. The authors also suggest the HFA was the most sensitive test for detecting the hearing loss induced by noise compared to other exams. The HFA is an important audiological exam for the early detection of hearing losses due to base lesions of the cochlear duct.^9^

In the clinical practice, it is possible to observe the need of monitoring patients with normal hearing associated with tinnitus. Data found in this study emphasizes the need of complementary exams as patients with normal hearing, based on the conventional protocol, are discharged and referred to counter-referral services.

Several authors observed that, in high frequencies, individuals with tinnitus presented hearing thresholds worse than individuals without tinnitus,^10^, ^11^, ^12^ corroborating this study data, which indicates the impairment of the hearing acuity at high frequencies.

In this study, G2 patients were satisfied with the adjustments from the monitoring, programming and individual specific needs. The HA is usually beneficial for patients with tinnitus that also have a significant hearing loss. Some patients confuse the origins of the deficits and blame the tinnitus for their communication difficulties, which are initially caused by hearing loss and, possibly, with the addition of the tinnitus. That is understandable as the hearing loss often progresses slowly over time and people not always realize they are losing acuity. In this context, study affirm the tinnitus is the addition of an unpleasant perception with a subtle beginning, and that most patients pay more attention to the tinnitus in daily life than to their gradual hearing loss.^3^

Therefore, the professional will be questioned and confronted about the intervention in several situations. Many patients will focus on the tinnitus as this is what really bothers them, not giving importance to the hearing treatment. Thus, the professional has to know the case in details and clarify the facts beforehand. The patients must understand the relation between the hearing loss and the tinnitus, once the prognosis tends to be positive from this understanding.

The questionnaire THI is a key tool for checking the nuisance caused by tinnitus and assessing the benefit of the intervention.^5^ In this study, the THI helped to assess the impact of tinnitus in the individual's quality of life, enabling the observation of the clinical status evolution of the groups studied.

The overall sample of groups assessed in this study presented an average THI of 66.66 points for G1 and 66.40 for G2, which corresponds to a moderate degree of nuisance, motivating the individual to look for intervention regardless the hearing loss. The samples showed very close nuisance indexes, excluding the possibility of normally hearing patients presenting a greater nuisance with the symptom (Table 1).

Data suggests an improvement in the long term for individuals with normal hearing and, in the short term, for individuals with hearing loss (Table 1). Such improvement would be justified by the instantaneous and immediate modification of the condition of the auditory nervous system from the fulfillment of acoustic and sensory needs using the HA, with an enrichment of the environmental sound associated with the SG. That does not occur in individuals with preserved hearing thresholds as their possible hearing deficits are in areas more distant from the SG stimulus, demanding more time for the habituation and break of the vicious behavioral cycle due to the nuisance generated by the symptom.

The same interpretation occurs for the use of the VAS, which enables the verification of the tinnitus intervention in a more objective way, once the patient notices the improvement in a succinct and timely manner with visual aid. The groups presented a decrease of the scores in the three assessment situations during the study (Table 2).

In the results obtained by the THI, the nuisance of the Groups 1 and 2 was modified from moderate to discreet, with a reduction greater than 20 points for 100% of the patients. The results found in the VAS indicated a reduction from 8.66 to 2.93, and then 1.80 in G1, and from 8.00 to 3.40, and then 1.66 in G2, which reinforces the effectiveness of the treatment for the groups with the same pattern: an initial high improvement and the maintenance of the benefit with a discreet improvement until the final assessment.

Based on the THI, attested that most patients presented an insignificant or mild nuisance, and this symptom did not interfere in the daily activities. However, in the clinical routine, it is possible to find cases in which the symptom is very uncomfortable and disabling.^13^

The research of the tinnitus frequency indicates acute psychoacoustic thresholds (Table 3), not extending to higher frequencies, a factor that contributed to the satisfactory results after intervention. The prognosis is positive when the frequency of the tinnitus is within the limits of the frequency range of the HA selected for the adaptation.^14^ Corroborating this finding, the total sample was benefited with the use of the HA and SG, with a frequency range up to 8 kHz, favoring the symptom improvement.

We observed in the clinical practice that, after the partial or total suppression of the symptom, some patients requested to be discharged from treatment, as they were satisfied and unaware that the hearing difficulties would continue the same without the rehabilitation, and there could be recurrence of tinnitus due to the lack of sensory stimulation. In these situations, the professional must resume the initial guidance and alert the communication difficulties will continue if they do not use the HA and the tinnitus will be back it there is no sound stimulation to the brain. Study agree with this assumption, reporting the use of the HA to stimulate the hearing system may contribute with the permanent reduction of the neural activities suggestively responsible for the generation and perception of the tinnitus.^3^

In the present study, it was possible to show the effectiveness of the SG in the tinnitus treatment in the two groups. This finding was observed in the results that were positive and similar in both groups, measured by the THI and the VAS as a reference of the modification of the handicap. These findings corroborate the study that verified an improvement in the tinnitus with the HA, HA associated with SG and the SG, with no superiority among the devices.^15^

Regarding the stimulus used for the groups, the noise provided was the “White Noise”, characterized by the sample as a comfortable wheezing. Study suggested the wideband noise is more effective than the narrowband noise.^16^ Authors indicated the “Speech Noise” as more tolerable and more likely to suppress the tinnitus with more effectiveness; however, the objective of the study was not the comparison between these stimuli.^17^

This research found the least intensity capable of providing the relief of the symptom, as suggested in study.^6^ However, there is evidence the therapeutic approaches interfere in the result obtained by the intervention. Study comparing the effect of the tinnitus management with the mixing-point masking (total) proposed by the Tinnitus Retraining Therapy (TRT), reported the masking was more effective in the first three months and that both approaches equaled in the sixth month. TRT showed to be more effective in the twelfth month. This aspect is similar to the results of the present study, in which there were a more significant improvement in the first three months of intervention.^16^ It is worth to highlight that, regardless the clinical sound therapeutic approach selected, the intervention has its effect primarily associated with counseling.^6^, ^18^ Counseling helps in breaking the vicious cycle caused by the tinnitus, supporting the patients’ decision-making, as well as the coping and the behavior change, was observed that affirmed a greater effectiveness of the treatment from the use of the HA with counseling compared to the counseling only.^19^

The associated approach used in this study was very effective, and we measured the treatment success numerically. The values observed in the THI were 22.13 and 18.66 for Groups 1 and 2, respectively, a satisfactory improvement in the first three months of intervention. The same patterns continued in the next 6 months, and the indexes obtained in the 6-month reassessment were 11.6 points and 10.6 points. A proportionality was achieved between the groups with a qualitative leap of the first assessment in relation to the second, and a more discreet one in relation to the third, suggesting the effective intervention may be verified in the first three months.

According to the results, 3 months were enough to verify the benefit of the HA associated with the SG and SG isolated. Study obtained the reduction of the nuisance in the first months of intervention with the group they analyzed.^20^

Authors, on the other hand, compared the benefit of using the HA and the SG (isolated) in the reduction of the nuisance generated by the tinnitus during 12 months, and observed the improvement of the symptom occurred in a progressive way from the three months of intervention, without any difference among the groups.^15^

Specifically, the G2 analysis shows the HA, besides helping in the amplification of the environmental sounds and speech recognition, favors the stimulation of the central auditory system, providing the reduction of the tinnitus perception.

The present study verified the use of the SG for patients with or without hearing loss was equally effective in the reduction of the nuisance caused by the tinnitus. Thus, its effectiveness and need of indication for patients with normal hearing is emphasized in the absence of the symptom.

The audiological knowledge on the pathophysiology of the tinnitus associated with the clinical knowledge to assess both the symptom and its consequences and implications in the patient's overall quality of life is necessary. No technique or approach isolated is enough for the intervention and promotion of benefit results in a symptom with a significant subjectivity and individuality.

## Conclusion

The present study enabled us to conclude the sound generator associated with the counseling was effective in the treatment of the tinnitus in individuals with and without hearing loss, providing the improvement of the nuisance and handicap.

## Conflicts of interest

The authors declare no conflicts of interest.

## References

[1] Noreña A.J. (2011). An integrative model of tinnitus based on a central gain controlling neural sensitivity. Neurosci Biobehav Rev.

[2] Moffat G., Adjout K., Gallego S., Thai-Van H., Collet L., Noreña A.J. (2009). Effects of hearing aid fitting on the perceptual characteristics of tinnitus. Hear Res.

[3] Folmer R.L., Carroll J.R. (2006). Long-term effectiveness of ear-level devices. Otolaryngol Head Neck Surg.

[4] Santos G.M., Bento R.F., Medeiros I.R.T., Oiticcica J., Silva E.C., Penteado S. (2014). The influence of sound generator associated with conventional amplification for tinnitus control: randomized blind clinical trial. Trends Hear.

[5] Ferreira P.E.A., Cunha F., Onishi E.T., Branco F.C.A., Ganança F.F. (2005). Tinnitus Handicap Inventory: adaptação cultural para o português brasileiro. Pró-Fono.

[6] Tyler R.S., Tyler R.S. (2006). Tinnitus treatment: clinical protocols.

[7] Noreña A.J., Tomita M., Eggermont J.J. (2003). Neural changes in cat auditory cortex after a transient pure-tone trauma. J Neurophysiol.

[8] Mehrparvar A.H., Mirmohammadi S.J., Davari M.H., Mostaghaci M., Mollasadeghi A., Bahaloo M. (2014). Conventional audiometry, extended high-frequency audiometry, and DPOAE for early diagnosis of NIHL. Iran Red Crescent Med J.

[9] Kleinberg K.F., Oliva F.C., Gonçalves C.G.O., Lacerda A.B.M., Garofani V.G., Zeigelboim B.S. (2011). High-frequency audiometry in audiological complementary diagnosis: a revision. Rev Soc Bras Fonoaudiol.

[10] Azevedo L.L., Iório M.C.M. (1999). Estudo dos limiares de audibilidade nas altas frequências em indivíduos de 12 a 15 anos com audição normal. Acta AWHO.

[11] Figuerêdo R.B.S., Corona A.P. (2007). Tinnitus influence on high-frequency hearing thresholds. Rev Soc Bras Fonoaudiol.

[12] Shim H.J., Kim S.K., Park C.H., Lee S.H., Yoon S.W., Ki A.R. (2009). Hearing abilities at ultra-high frequency in patients with tinnitus. Clin Exp Otorhinolaryngol.

[13] Carvalho R.C., Souza R.T., Silva M.S., Souza J.A. (2010). Uso do questionário Tinnitus Handicap Inventory adaptado para o português para avaliação da qualidade de vida dos pacientes com queixa de zumbido atendidos no Ambulatório Araújo Lima. Revhugv.

[14] McNeill C., Tavola-Vieira D., Alnafjan F., Searchfield G., Welch D. (2012). Tinnitus pitch, masking, and the effectiveness of hearing aids for tinnitus therapy. Int J Audiol.

[15] Parazzini M., Del Bo L., Jastreboff M., Tognola G., Ravazzani P. (2011). Open ear hearing aids in tinnitus therapy: an. efficacy comparison with sound generators. Int J Audiol.

[16] Henry J.A., Schechter M.A., Zaugg T.L., Griest S., Jastreboff P.J., Vernon J.A. (2006). Clinical trial to compare tinnitus masking and tinnitus retraining therapy. Acta Otolaryngol Suppl.

[17] Ito M., Soma K., Ando R. (2009). Association between tinnitus retraining therapy and tinnitus control instrument. Auris Nasus Larynx.

[18] Jastreboff P.J. (1990). Phantom auditory perception (tinnitus): mechanisms of generation and perception. Neurosci Res.

[19] Searchfield G. (2011). A commentary on the complexity of tinnitus management clinical guidelines provide a path through the fog. Eval Health Prof.

[20] Ferrari G.M.S., Sanchez T.G., Pedalini E.B.A. (2007). eficácia do molde aberto para o controle do zumbido. Braz J Othorhinolaryngol.

